# Recent Progress in Intrinsically Stretchable Sensors Based on Organic Field-Effect Transistors

**DOI:** 10.3390/s25030925

**Published:** 2025-02-04

**Authors:** Mingxin Zhang, Mengfan Zhou, Jing Sun, Yanhong Tong, Xiaoli Zhao, Qingxin Tang, Yichun Liu

**Affiliations:** Center for Advanced Optoelectronic Functional Materials Research, Key Laboratory of UV-Emitting Materials and Technology of Ministry of Education, Northeast Normal University, Changchun 130024, China; zhangmx539@nenu.edu.cn (M.Z.); mengfanzhou@nenu.edu.cn (M.Z.); sunj545@nenu.edu.cn (J.S.); zhaoxl326@nenu.edu.cn (X.Z.); ycliu@nenu.edu.cn (Y.L.)

**Keywords:** intrinsically stretchable sensors, organic field-effect transistors (OFETs), sensing mechanism, sensing devices

## Abstract

Organic field-effect transistors (OFETs) are an ideal platform for intrinsically stretchable sensors due to their diverse mechanisms and unique electrical signal amplification characteristics. The remarkable advantages of intrinsically stretchable sensors lie in their molecular tunability, lightweight design, mechanical robustness, solution processability, and low Young’s modulus, which enable them to seamlessly conform to three-dimensional curved surfaces while maintaining electrical performance under significant deformations. Intrinsically stretchable sensors have been widely applied in smart wearables, electronic skin, biological detection, and environmental protection. In this review, we summarize the recent progress in intrinsically stretchable sensors based on OFETs, including advancements in functional layer materials, sensing mechanisms, and applications such as gas sensors, strain sensors, stress sensors, proximity sensors, and temperature sensors. The conclusions and future outlook discuss the challenges and future outlook for stretchable OFET-based sensors.

## 1. Introduction

Intrinsically stretchable sensors have garnered considerable attention due to their promising applications in wearable electronics, healthcare monitoring, and human–machine interfaces for robotics [1,2,3,4,5,6,7,8,9,10]. Compared with conventional rigid sensors, these sensors can be stretched, bent, or compressed while retaining their electrical functionality under deformation [11,12]. Among the various stretchable sensors, organic field-effect transistor (OFET)-based sensors are particularly noteworthy for their exceptional sensitivity, rapid response times, and ability to integrate multiple sensing modalities into a single device. This versatility makes them ideal candidates for the real-time multimodal detection of a broad range of signals and stimuli, such as gasses, pressure, strain, proximity, and temperature.

**Figure 1:**
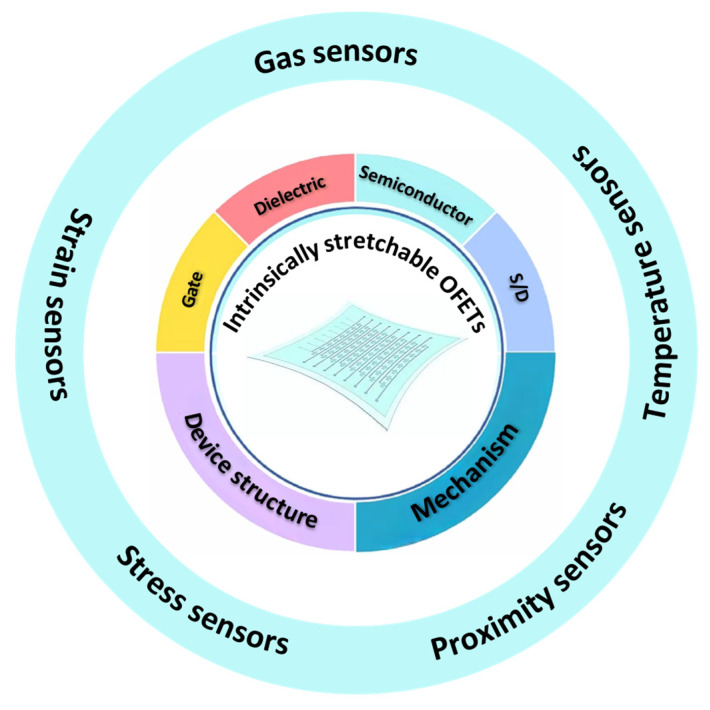
Schematic illustration of intrinsically stretchable OFET-based sensors.

Two main approaches have been applied in stretchable OFETs, namely structural design and intrinsically stretchable materials. Compared with structurally designed stretchable OFETs, intrinsically stretchable OFETs offer advantages such as high integration, low cost, superior mechanical robustness, and excellent biocompatibility. Intrinsically stretchable OFETs are composed of source–drain electrodes, gate electrodes, semiconductor layers, and dielectric layers (Figure 1). Multiple interfaces are formed between these components, including the source–drain electrode/semiconductor interface, semiconductor/dielectric layer interface, dielectric layer/gate electrode interface, and the semiconductor/air interface [13]. When an OFET-based sensor interacts with an analyte, various factors, including the composition, stacking configuration, and charge density of the active material, as well as properties such as interface polarity, resistance, potential, and ion migration, are modulated [14]. These changes directly influence the output electrical signals, including source–drain currents (*I*_DS_), threshold voltage (*V*_T_), the on/off ratio, and subthreshold swing (SS). The interplay of multiple components, interfaces, and electrical parameters enables OFET-based sensors to seamlessly integrate diverse sensing functions, positioning them as a versatile platform for multifunctional sensing applications. However, the scarcity of stretchable materials and the stability of electrical performance under high strains remain key challenges for intrinsically stretchable OFET-based sensors.

In this review, we focus on the recent progress of intrinsically stretchable sensors based on OFETs from functional materials, sensing mechanisms, and sensing applications, such as gas sensors, strain sensors, stress sensors, proximity sensors, and temperature sensors. Finally, we summarize our findings in the Conclusion and provide the future outlook for the intrinsically stretchable sensors based on OFETs.

## 2. Operating Mechanism of Intrinsically Stretchable OFETs

**Figure 2:**
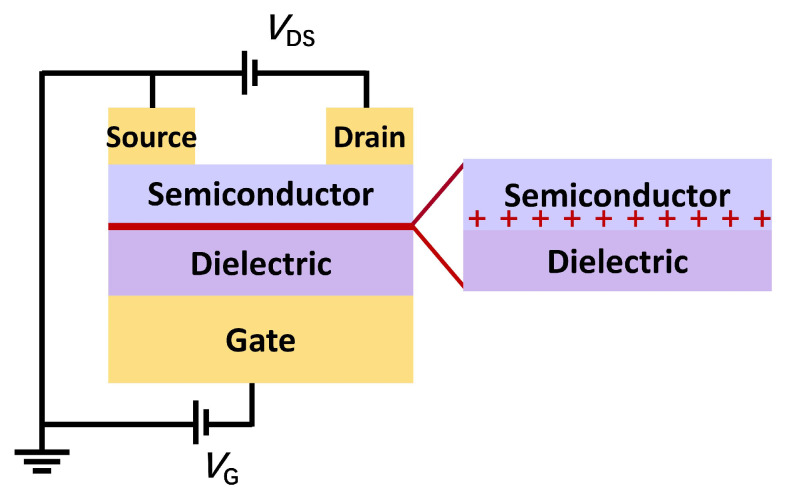
Schematic diagram of OFET structure and operating mechanism.

The stretchable OFETs can be modeled as parallel plate capacitors, where the conductive channel of the semiconductor layer serves as the upper plate and the gate electrode acts as the lower plate (Figure 2). When a gate voltage is applied, electrostatic induction causes charge carriers to accumulate in the conductive channel. These induced charge carriers participate in conduction, forming a conductive channel and generating a source–drain current. Typically, the carrier concentration in organic semiconductors is pretty low. When no gate voltage is applied, the semiconductor exhibits high resistivity and low currents. However, when the gate voltage is applied, the charge carrier concentration in the conductive channel increases sharply, reducing the resistivity of the semiconductor layer and enabling the formation of a source–drain current (*I*_DS_). Consequently, the gate is often metaphorically referred to as a “valve” that controls the current in the conductive channel, similar to a faucet that controls the flow of water.

**Figure 3:**
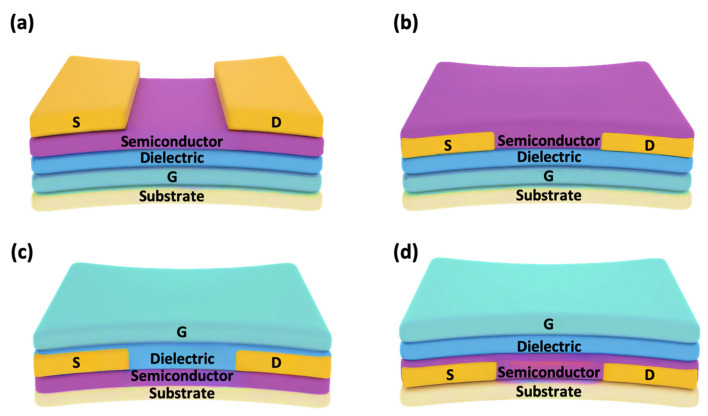
Four typical device structures of stretchable OFETs, including (**a**) bottom-gate top-contact structure, (**b**) bottom-gate bottom-contact structure, (**c**) top-gate top-contact structure, and (**d**) top-gate bottom-contact structure. (S: source electrode, D: drain electrode, G: gate electrode).

Typically, a stretchable OFET consists of five components, namely the source electrode(S), drain electrode(D), semiconductor layer, insulator layer, and gate electrode(G). Based on the position of the semiconductor and electrodes, they can be classified as top-contact and bottom-contact configurations. For the same organic semiconductor material, top-contact OFETs often exhibit superior performance to bottom-contact OFETs. This can be attributed to two key factors: first, the top-contact devices have larger injection areas than bottom-contact OFETs, thereby reducing the contact resistance between the electrode and the semiconductor layer; second, the top-contact OFETs provide an improved semiconductor/electrode interface, further minimizing contact resistance. However, the top-contact structure is less compatible with large-scale production processes, posing a significant limitation to the practical application of top-contact OFETs. There are four basic configurations of stretchable OFETs based on the position of the gate electrode and source/drain electrodes, including bottom-gate top-contact (BGTC) (Figure 3a), bottom-gate bottom-contact (BGBC) (Figure 3b), top-gate top-contact (TGTC) (Figure 3c), and top-gate bottom-contact (TGBC) (Figure 3d). Among these four basic OFET configurations, the bottom-gate bottom-contact OFETs are more suitable for sensor devices, as the conductive channel is exposed to the target atmosphere, leading to enhancing or reducing device conductivity by inducing or capturing additional charges. The changes in the conductivity of OFETs lead to variations in the basic parameters such as threshold voltage, field-effect mobility, and source–drain currents, thereby generating the output signals of the sensors.

## 3. Materials for Intrinsically Stretchable OFETs

**Figure 4:**
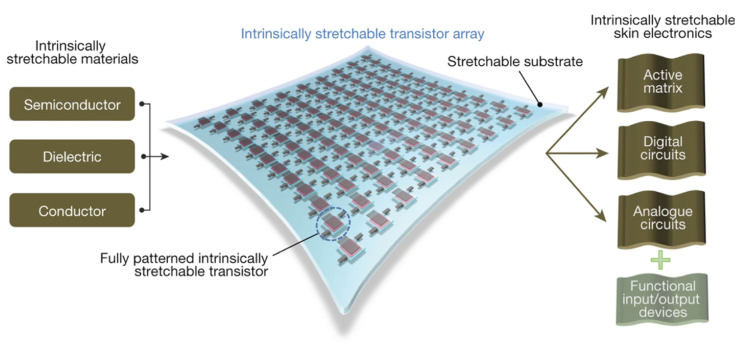
Schematic illustration of an intrinsically stretchable transistor, including intrinsically stretchable conductor, semiconductor, and dielectric materials. Reproduced with permission from [20], copyright 2018, Springer Nature.

Human skin, tissues, and organs are soft and constantly in motion, leading to high deformation. For instance, the cerebral cortex can undergo deformation of approximately 10%, the pulsating heart by around 20~35%, the knee by roughly 49%, the elbow by up to 60%, and the highly flexible finger joints can deform as much as 80%. As a result, traditional flexible transistors face significant challenges in meeting the demands at high strain [15]. The main challenge in stretchable electronics is ensuring that materials can withstand mechanical strains while maintaining high electrical performance. Designing mechanical structures such as wavy structures [16] and interconnected rigid island bridge structures [17] can improve the device stretchability. However, the stretchable devices using this method exhibit low conformability, poor biocompatibility, low device integration, complex manufacturing processes, and reduced transparency [18,19,20]. As mentioned above, intrinsically stretchable OFETs require that every component possesses excellent stretchability, such as conductor materials, semiconductor materials, and dielectric materials, thereby imposing stricter demands on the materials, as shown in Figure 4 [21]. Compared to structural design, the intrinsically stretchable OFETs offer several advantages, including higher device density, improved biocompatibility, lower fabrication complexity, and reduced cost. Moreover, the low Young’s modulus of these materials allows for seamless conformity to various surfaces, enhancing comfort in human applications and improving signal fidelity [22].

However, fabricating intrinsically stretchable OFETs requires that every component possesses excellent stretchability, which imposes stricter requirements on the materials. The biggest challenges are the scarcity of the stretchable semiconductor materials and the limitations of device fabrication and integration technologies [21]. For example, stretchable OFETs require elastic polymers as insulating layers and substrates. Unfortunately, the lithography techniques in the microelectronics industry are incompatible with these polymers, as the solvents and ultraviolet light used in lithography damage elastic polymers, leading to misaligned multilayer structures and malfunctioning devices. Therefore, advancing research into intrinsically stretchable materials and optimizing fabrication processes for these devices are crucial. These efforts will enhance the advantages of stretchable OFETs, accelerate the development of flexible electronics, and deepen researchers’ understanding of this field.

### 3.1. Intrinsically Stretchable Conductive Materials

Stretchable electrodes are key components of stretchable OFETs. The stretchability and electrical properties are critical parameters for stretchable electrodes. There are two main strategies for intrinsically stretchable electrodes, namely carbon materials (carbon nanotubes (CNTs), conducting polymers (poly(3,4-ethylenedioxythiophene):poly-(styrenesulfonate), PEDOT:PSS), and metallic nanowires. In this section, we will discuss the recent progress of intrinsically stretchable conductive materials.

#### 3.1.1. Carbon Materials for Stretchable Electrodes

Carbon nanotubes (CNTs) are traditional stretchable electrode materials in intrinsically stretchable OFETs. Carbon nanotubes (CNTs) are nanoscale hollow coaxial tubes formed by curling one or more graphene sheets. The interlayer spacing is approximately 0.34 nm, with diameters ranging from 2 to 20 nm and lengths extending up to several meters. This high aspect ratio confers exceptional stretchability to CNTs, enabling the formation of large-pore permeation networks that enhance the transmittance of visible light. In addition, CNTs exhibit remarkable mechanical properties, with a Young’s modulus of around 1 TPa and a tensile modulus of 140 GPa alongside excellent electrical conductivity (1 × 10^8^ S/m) [23]. Furthermore, CNTs can be produced in large quantities at relatively low cost, making them an attractive conductive material for the fabrication of flexible electrodes.

**Figure 5:**
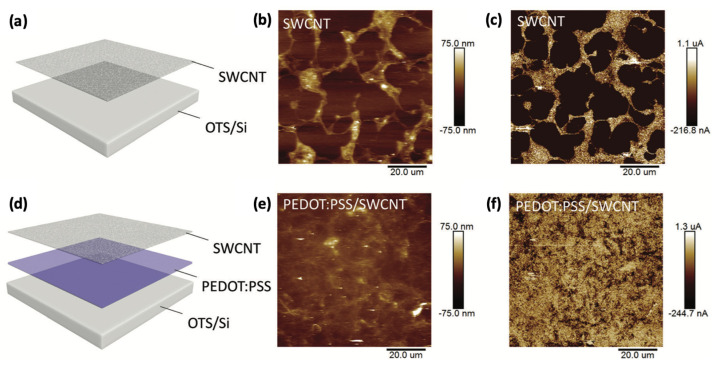
Schematic illustration, AFM, and conductive AFM images of intrinsically stretchable electrodes of (**a**–**c**) SWCNT and (**d**–**f**) PEDOT:PSS/SWCNT. Reproduced with permission from [25], copyright 2019, RSC publishing.

Bao et al. [24] reported intrinsically stretchable OFETs with a bottom-gate bottom-contact structure and a CNT as the electrode, polystyrene-block-poly(ethylene-ran-butylene)-block-polystyrene (SEBS) as the dielectric and substrate, and poly(2,5-bis(2-octyldodecyl)-3,6-di(thiophen-2-yl)diketopyrrolo[3,4-c]pyrrole-1,4-dione-alt-thieno[3,2-b]thiophen) (DPPT-TT)/SEBS as the semiconductor. The fabricated OFETs have good transparency and excellent conformability on human skin. The average mobility of 20 devices is 0.59 cm^2^ V^−1^ s^−1^, even when the device is stretched to 100% without affecting the mobility, which is comparable to the amorphous silicon. Tang et al. [25] reported a novel photolithographic stretchable CNT/PEDOT:PSS hybrid electrode, as shown in Figure 5. The PEDOT:PSS provides a highly wettable interface for the CNT aqueous solution, enabling the formation of a uniform conductive film that achieves high-precision electrode patterns down to 3 µm, high conductivity even when stretched up to 100%, and high transparency up to 84% at 550 nm.

#### 3.1.2. Conducting Polymers for Stretchable Electrodes

Conductive polymers can not only alter mechanical and electrical properties by regulating their molecular structure, but they are also easy to process, making them promising candidates for the preparation of elastic conductors. Poly(3,4-ethylenedioxythiophene): poly(styrene sulfonate) (PEDOT:PSS) exhibits very high conductivity (1000 S/m) and a Young’s modulus greater than 2 GPa, with optical transmittance comparable to that of ITO. As a result, it has become one of the most commonly used conductive polymers [26]. However, its low elongation (approximately 10%) [27] has seriously hindered the development of PEDOT:PSS in stretchable electrodes.

**Figure 6:**
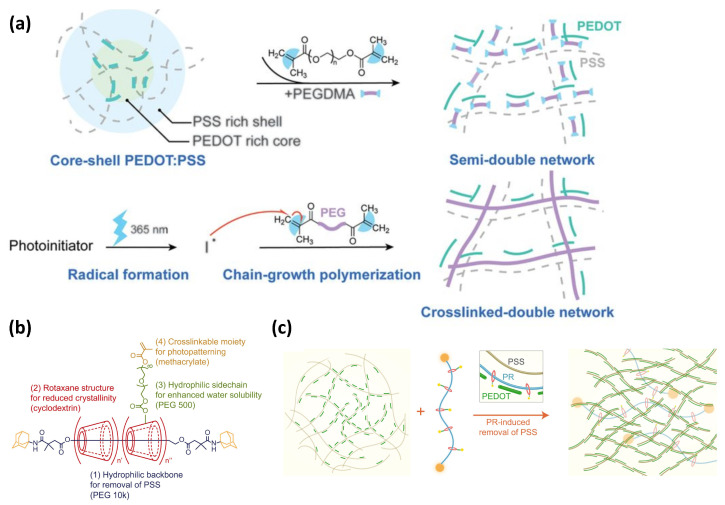
(**a**) Mechanism of lithography of PEDOT:PSS and PEGDMA. Reproduced with permission from [28], copyright 2021, AAAS Science. (**b**) Chemical structure of PR-PEGMA. (**c**) Schematic diagram illustrating PR and PEDOT:PSS for enhanced conductivity. Reproduced with permission, copyright [29] AAAS Science.

In 2021, Bao et al. [28] reported that the use of PEDOT:PSS with the crosslinker polyethylene glycol dimethacrylate (PEGDMA) for direct optical lithography patterning, with feature sizes down to 2 μm, high conductivity of 52.5 KS/m, and high stretchability up to 100% (Figure 6a). In 2022, they [29] developed a molecular engineering strategy based on PEDOT:PSS and polyrotaxane (PR) topological supramolecular networks (Figure 6b,c), achieving the three following important breakthroughs: (i) stretchable conductive polymers with high conductivity and biocompatibility, (ii) direct photopatterning with feature sizes as small as the cellular level (about 2 μm), and (iii) maintaining high stretchability after micro-nanofabrication, with no crack formation under 100% strain.

#### 3.1.3. Metallic Nanowires for Stretchable Electrodes

Metallic nanowires, such as silver nanowires (AgNWs) and gold nanowires (AuNWs), are typical stretchable conductive materials due to their high conductivity and stretchability. These nanowires, with low sheet resistance (<50 Ω/sq), high transmittance (>90%), and high aspect ratios, enable high conductivity under high strains [30]. Lee et al. [31] prepared fully stretchable OFETs based on AgNWs. The sprayed AgNWs were used as the source/drain electrodes for the OFETs. The OFETs prepared in this way maintained a hole mobility of about 0.1 cm^2^ V^−1^ s^−1^ at a low operating voltage of 3 V, even when stretched to 80% strain. Zhu et al. [32] reported intrinsically stretchable vertical AuNW electrodes for OFETs. The vertical growth of AuNWs was successfully achieved using a seeded growth method at room temperature. Even under a strain of 170%, the conductivity of the vertical AuNWs remained as high as 152 S cm^−1^. Stretchable OFETs fabricated with AuNWs exhibited stable performance, with only minimal degradation when subjected to up to 100% strain.

### 3.2. Intrinsically Stretchable Semiconductor Materials

So far, many reports have focused on stretchable electrode materials, but semiconductor materials that combine good electrical properties with stretchability are still relatively scarce. The lack of intrinsically stretchable semiconductor materials has significantly hindered the development of intrinsically stretchable OFETs. Most flexible OFETs are made from small-molecule semiconductor materials, which have simple structures, high crystallinity, ease of processing, and good electrical properties. However, their strain range is typically limited to 5–10% [33]. In contrast, polymer semiconductor materials generally have a lower elastic modulus (in the range of hundreds of MPa), are compatible with solution processing, and offer distinct advantages for developing intrinsically stretchable electronic materials. Polymer molecular chains exist in various forms, such as folded chains, loose rings, suspended segments, and entangled chains. As a result, most polymers are composed of both ordered (crystalline regions) and disordered (amorphous regions) domains [34]. Ordered regions play a crucial role in charge transport in high-performance polymer semiconductors. Charges are typically carried along the connecting molecules of adjacent ordered aggregates or individual polymer chains, moving from one part of the ordered region to another. Conversely, disordered regions create high-energy barriers, which significantly hinder charge transport. Consequently, efficient charge transport usually requires highly crystalline conjugated polymer films, while good mechanical properties demand low crystallinity. These two conflicting requirements make the design of high-mobility intrinsically stretchable polymer semiconductor materials a recognized challenge in the field [35]. At present, common stretchable polymer semiconductor materials are divided into three categories, namely poly(3-hexylthiophene) (P3HT) [36,37], diketopyrrolopyrrole (DPP) conjugated polymers [18,19,38,39], and nanoconfinement effect films. However, more intrinsically stretchable semiconductor materials need further exploration.

#### 3.2.1. Poly(3-Hexylthiophene) (P3HT)

DeLongchamp et al. [36] were the first to highlight that variations in molecular structure significantly influence the mechanical properties of polymers. In their comparative study of P3HT and PBTTT, they demonstrated that P3HT, with moderate crystallinity, exhibits low charge mobility, whereas PBTTT, characterized by high crystallinity, exhibits high mobility. P3HT adopts a conjugated layered stacking arrangement, separated by alkyl side chains, with its conjugated backbone organized in an “edge-on” configuration, aligning the π-π plane parallel to the substrate. In contrast, PBTTT, possessing higher charge mobility, forms a three-dimensional crystalline structure due to the staggered alignment of its alkyl side chains, resulting in larger grain sizes and reduced defect density. This structural distinction also accounts for the disparity in the elastic modulus, with 0.25 GPa for P3HT versus 0.9 GPa for PBTTT. Consequently, PBTTT has a crack initiation strain of only 2.5% compared to 150% for P3HT. Similar observations have been reported by other research groups, reinforcing the notion that the excellent electrical and mechanical properties of conjugated polymers are inherently conflicting. From a morphological perspective, achieving high electrical performance necessitates high crystallinity, while good mechanical properties require the presence of amorphous regions. As a result, P3HT has emerged as a widely studied model material for stretchable polymer semiconductors due to its commercial availability and well-documented properties.

#### 3.2.2. Diketopyrrolopyrrole (DPP)-Conjugated Polymers

Wu et al. conducted the first investigation into the carrier mobility of two donor–acceptor (D-A) type polymers, PiI-2T and PDPP-FT4, under tensile strain [40]. Their findings revealed that the stretchability of PiI-2T closely resembles that of P3HT, maintaining a mobility of 1.52 × 10^−2^ cm^2^ V^−1^ s^−1^ under 100% tensile strain. In contrast, the mobility of PDPP-FT4 declined significantly as tensile strain increased.

Roth et al. explored a series of D-A polymers with varying molecular structures, uncovering that despite similar elastic moduli to P3HT, most D-A polymers exhibited lower initial crack strains and displayed brittle behavior. Their results indicated that polymer backbones containing multiple rigid fused-ring structures are generally more brittle than those with single-ring structures, whereas polymers with longer branched side chains tend to exhibit greater ductility [41].

Recently, Bao’s group introduced a novel approach to enhance the tensile properties of conjugated polymer films by designing molecular structures with conjugated blocking regions containing dynamic non-covalent cross-linking units. In their earlier work [42], they demonstrated that incorporating multiple dynamic cross-linking sites into the polymer backbone facilitates various energy dissipation mechanisms. Building on this concept, they synthesized a series of conjugated polymer semiconductors by introducing PDCA units that feature moderate hydrogen bonding forces into the DPP molecular structure [18]. Compared to PDPP-TVT molecules without PDCA units, the modified polymers exhibited a reduced elastic modulus (from 1 to 0.25 GPa) and a significantly improved crack initiation strain of approximately 120%. This reduction in the elastic modulus was attributed primarily to the shortened conjugation length. Among these polymers, P3 maintained a mobility exceeding 1 cm^2^ V^−1^ s^−1^ under 100% tensile strain, with the mobility decreasing by only 26% after 100 cycles of 100% stretching. The enhanced stretchability was largely ascribed to the sacrificial nature of the weak hydrogen bonds in the PDCA units, which break under strain to relieve stress on the polymer backbone. To validate this hypothesis, the researchers methylated the amide groups in PDCA to eliminate hydrogen bonding (P6) and re-evaluated its mechanical properties. Although the elastic modulus decreased due to the presence of conjugation blocking regions, the crack initiation strain dropped significantly to just 25%, highlighting the critical role of hydrogen bonding in enhancing stretchability. Furthermore, the dynamic nature of hydrogen bonds enabled P3 molecules to self-heal in the presence of a solvent or upon heating after being damaged by stretching. Post-repair, the cracks became nearly invisible, and the mobility was restored to 1 cm^2^ V^−1^ s^−1^.

In addition to investigating the intrinsic stretchability of polymer semiconductors, some research groups have explored blending these materials with other semiconductors or insulators exhibiting superior stretchability to enhance their mechanical properties. O’Connor et al. [43] blended the high-performance conjugated polymer PCDTPT with high-molecular-weight P3HT, a material known for its excellent stretchability. Without blending, PCDTPT exhibited an average mobility of 0.9 cm^2^ V^−1^ s^−1^ but developed significant cracks at a tensile strain of just 4%. When mixed with P3HT at a 1:1 ratio, the composite film could endure strains up to 75%, and its mobility increased to 1.2 cm^2^ V^−1^ s^−1^, potentially due to molecular rearrangements induced by tensile stress.

Choi et al. conducted detailed studies on a system combining self-assembled P3HT nanofibers with PDMS. P3HT nanofibers were synthesized through sonication and the use of a poor solvent (2-methylpentane), enabling fiber growth from a supersaturated P3HT solution upon cooling. The mobility of P3HT fibers without PDMS was limited to 0.088 cm^2^ V^−1^ s^−1^. However, when 0.75% P3HT was blended with PDMS, the mobility increased to 0.18 cm^2^ V^−1^ s^−1^. Under 100% tensile strain, the composite film exhibited no visible cracks, although the authors did not report its electrical properties under varying strains [44].

Shin et al. developed a composite by cooling a P3HT/SEBS mixed solution at −15 °C, followed by spin-coating the resulting fibers onto a PDMS substrate [45]. XPS analysis revealed that most P3HT fibers were concentrated near the top surface of the film, with a smaller portion distributed at the bottom. Mechanical testing demonstrated an elastic modulus as low as 11.7 MPa and a fracture strain of 300%, comparable to that of pure SEBS films. When subjected to 50% tensile strain, the mobility decreased from 0.6 × 10^−2^ to 0.2 × 10^−2^ cm^2^ V^−1^ s^−1^. Notably, the electrical properties remained stable after 50 stretching cycles at 50% strain, with no significant degradation observed even after 200 cycles.

The aforementioned studies primarily focus on P3HT fibers pre-grown prior to film deposition. While these fibers exhibit high crystallinity, they are inherently limited by poor ductility. Additionally, the inclusion of insulating elastomers complicates the formation of robust nanostructural networks, resulting in suboptimal electrical performance. The primary failure mechanism in polymer semiconductor/insulator blend devices lies in the fracture of polymer fibers under low strain, as their ductility is constrained by the capacity of the fibers to reorient within the elastomer matrix.

#### 3.2.3. Nanoconfinement Effect

Bao et al. addressed this limitation by employing nanoconfinement to reduce the crystallinity and rigidity of polymer semiconductor fibers [19,20,21]. Nanoconfinement, a well-established approach for modulating ductility, molecular chain dynamics, and elastic modulus in polymers, had not been previously applied to polymer semiconductors. Xu et al. from Bao’s group demonstrated the preparation of polymer films with enhanced mechanical and electrical properties by blending DPPT-TT semiconductors with 70% SEBS. Unlike P3HT fiber/insulating elastomer blends, the high electrical performance in this system does not rely on pre-grown high-crystallinity fiber bundles but instead emerges from the formation of nano-agglomerates. The close surface energy compatibility between DPPT-TT and SEBS facilitates the development of a nanophase-separated structure. The confinement effect of SEBS suppresses polymer crystallization and lowers the glass transition temperature (Tg) of the polymer, resulting in reduced elastic modulus, increased crack initiation strain, and higher yield strain. Remarkably, while nearly all stretchable semiconductors experience a decline in mobility under tensile strain, this blended film can withstand 100% tensile strain without exhibiting significant cracking or mobility degradation. Moreover, the mobility remains stable even after 1000 cycles at 25% tensile strain, representing a significant advancement in the development of stretchable semiconducting materials.

### 3.3. Intrinsically Stretchable Insulator Materials

In addition to the scarcity of semiconductor materials, research on intrinsically stretchable insulating layer materials is also still in its infancy. Stretchable insulator materials mainly include the commercial elastomers PDMS and SEBS (Table 1). However, their low dielectric constant (2.7 and 2.1) leads to high operating voltage (−50~−100 V), which is much higher than the absolute value of human safety voltage (12 V) [46], leading to high device power consumption.

**Figure 7:**
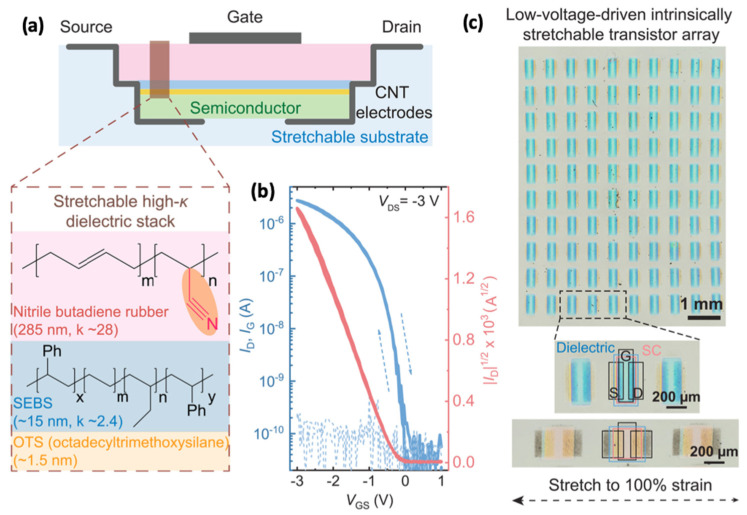
**Low-voltage stretchable OFETs.** (**a**) Device structure and tri-layer insulators. (**b**) Typical transfer curves of the OFETs. (**c**) Optical image of the low-voltage stretchable OFET array. Reproduced with permission from [63], copyright 2023, AAAS Science.

To improve safety and decrease the power consumption of OFETs, Bao’s group applied high-dielectric-constant (~28) nitrile–butadiene rubber (NBR) in intrinsically stretchable OFETs (Figure 7). However, the high-dielectric-constant elastomer suffers from energetic disorders at semiconductor–dielectric interfaces, leading to low carrier mobility and low stability. The trade-off between low operating voltage and high carrier mobility is challenging in stretchable OFETs. To overcome this issue, they applied a tri-layer structure consisting of crosslinked high-k NBR, an ultrathin poly(styrene-ethylene-butylene-styrene) (SEBS) elastomer (15 nm), and a hydrophobic octadecyltrimethoxysilane (OTS) molecular modification. The resulting low-polarity surface enables the intrinsically stretchable OFET array (Figure 7b) with low operational voltage down to −3 V, high carrier mobility up to 2.01 cm^2^ V^−1^ s^−1^, and high stretchability up to 100% [63]. However, elastomers easily swell or even dissolve in solvents, making multilayer deposition challenging.

Very recently, Tang’s group presented a dielectric design by incorporating a flexible small-molecule diamine crosslinking agent 4-aminophenyl disulfide (APDS), into (poly(propylene glycol), tolylene 2,4-diisocyanate terminated (PPG-TDI)), resulting in a single-layer PUU elastomer with high-*k* and low surface polarity for intrinsically stretchable OFETs [15]. Compared with commercial elastomers, such as poly(dimethylsiloxane) (PDMS184), PDMS186, poly(styrene-b-butadiene-b-styrene) (SBS), SEBS, thermoplastic polyurethane (TPU), and poly(methyl methacrylate)-poly(*n*-butyl acrylate)-poly(methyl methacrylate) (PMMA-PnBA-PMMA), PUU elastomers as dielectrics of the stretchable organic transistors show outstanding advantages, including lower surface roughness (0.33 nm), higher adhesion (45.18 nN), a higher dielectric constant (13.5), and higher stretchability (896%). The PUU dielectric allows the intrinsically stretchable, all-solution-processed organic transistor to function at an operational voltage as low as −10 V while maintaining a high mobility of 1.39 cm^2^ V^−1^ s^−1^. Notably, the transistor exhibits excellent electrical stability after 10,000 switching cycles and outstanding mechanical robustness, even when stretched up to 100%. This work introduces a novel molecular engineering method for achieving low-voltage, high-mobility, and stretchable all-solution-processed OFETs

## 4. Mechanism of OFET-Based Stretchable Sensors

When the analyte interacts with a component or interface of the stretchable OFETs, it induces changes in the charge concentration or transport properties of the OFETs, generating a corresponding electrical signal and completing the sensing process. OFETs possess multiple components, interfaces, and parameters, leading to diverse sensing mechanisms. The sensing mechanisms of OFETs can be categorized into the four types described below.

### 4.1. Interaction with the Semiconductor Layer

The analyte may interact with the organic semiconductor, altering its aggregation structure or charge concentration, which leads to changes in the electrical performance of the OFETs. Specific modes of interaction with the aggregation structure include solvent-induced swelling of the semiconductor film, stress or strain-induced changes in the semiconductor lattice, and temperature-induced deformation or phase transitions in the film. Regarding charge concentration, interactions such as charge transfer between redox-active gasses and the semiconductor, changes in potential barriers at grain boundaries, charge transfer between analytes and the conductive channels, or local potential disorders induced by polar molecules can all influence device behavior.

### 4.2. Contact Resistance Changes in Electrode

The total resistance between the source and drain electrodes of an OFET is a combination of the contact resistance at the electrode/semiconductor interface and the resistance of the conductive channel. This relationship can be expressed as

$$R_{total}=R_{con}+R_{ch}$$

In this equation, $R_{total}$, $R_{con}$, and $R_{ch}$ represent the total, contact, and channel resistances, respectively. When the OFETs experience pressure or material alterations, the contact resistance changes, leading to a change in the source–drain current of the OFETs.

### 4.3. Capacitance Changes in the Dielectric

Under the influence of the analyte, the dielectric capacitance changes, affecting the charge concentration induced and accumulated in the channel. As mentioned above, the OFETs can be modeled as parallel plate capacitors, with the gate electrode and the conductive channel serving as the two electrodes of the capacitor. According to the following equation:

$$C_{i}=\frac{{\varepsilon \varepsilon }_{0}}{d}$$

In this equation, $\varepsilon_{0}$ is the vacuum dielectric constant, ε is the relative dielectric constant of the dielectric layer, and d is the dielectric thickness. Under changes in pressure or temperature, the dielectric constant and the dielectric thickness will change, which will influence the dielectric capacitance, leading to changes in the channel charge concentrations and the electrical performance.

### 4.4. Potential Changes at the Gate Electrode/Dielectric Interface

Changes in the potential at the gate electrode/dielectric layer interface affect the effective gate voltage applied to the conductive channel. This mechanism is particularly prevalent in ion sensors, where it is referred to as the ion-sensitive OFET effect. In these OFETs, the traditional metal gate electrode is replaced by reference electrodes and electrolyte solutions. A gate voltage is applied through the reference electrode, but the effective gate voltage acting on the channel is influenced by the potential at the interface between the electrolyte solution and the dielectric layer, which is highly sensitive to the charge and concentration of the ions present.

## 5. Stretchable Sensors Based on OFETs

### 5.1. Stretchable Gas Sensors Based on OFETs

Toxic gasses pose serious risks to human health and environmental safety, making the fabrication of highly sensitive gas sensors that can distinguish between the types and concentrations of toxic gasses particularly important. Traditional metal oxide-based sensors have been widely used in gas sensing due to their high sensitivity and good stability. However, their requirement for high temperature and pressure not only increases energy consumption but is also incompatible with flexible and portable devices. In addition, their sensing parameters are relatively simple, the selectivity is not ideal, and the relatively large size also limits the device integration. In contrast, gas sensors based on OFETs exhibit many advantages of organic semiconductors, such as tunable molecular structures, solution processability, and mechanical flexibility, which enable the development of diverse and low-cost flexible electronic devices. Moreover, the multiple parameters of OFET-based sensors demonstrate great potential for applications in the next generation of wearable electronic devices. When an OFET is exposed to the target gas atmosphere, the gas molecules penetrate the semiconductor layer, resulting in either an enhancement or reduction in its channel conductivity. However, the limited availability of stretchable polymer semiconductor materials has led to the scarcity of reports on stretchable gas sensors based on OFETs.

**Figure 8:**
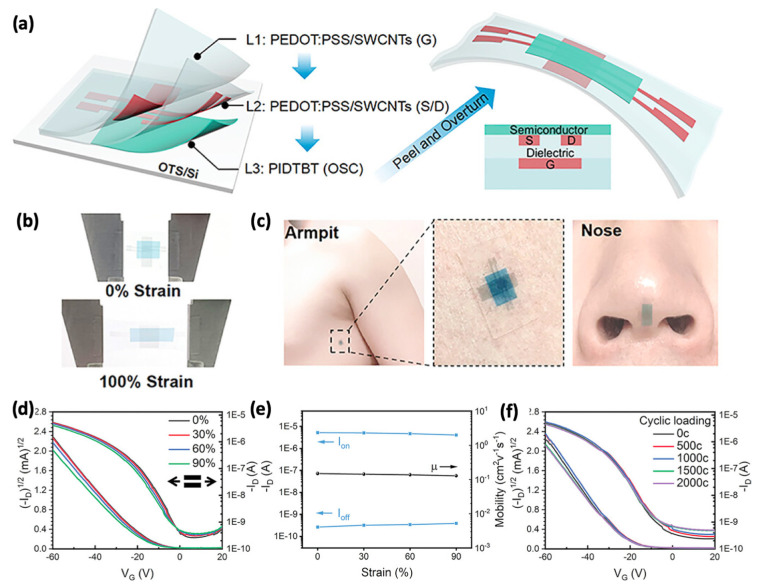
(**a**) Fabrication illustration of intrinsically stretchable transistor-based gas sensors. (**b**) Photographs of the fully intrinsically stretchable OFET-based sensors stretched to 100% strain. (**c**) The excellent conformability of the OFET-based sensors can conform on human skin. (**d**,**e**) Typical transfer curves, on and off currents, and mobilities under 0–90% strains. (**f**) The transfer characteristics of stretchable sensors show mechanical robustness, as the device can function well under 30% strain for 2000 cycles. Reproduced with permission from [67], copyright 2013, John Wiley and Sons.

The only reported intrinsically stretchable OFET-based gas sensors by Tang’s group [67] consist of intrinsically stretchable electrodes (PEDOT:PSS/SWCNT), an intrinsically stretchable semiconductor (PIDTBT), and an intrinsically stretchable insulator (PDMS), as shown in Figure 8a. In Figure 8b, the intrinsically stretchable gas sensors can be stretched to 100% without obvious damage. The stretchable gas sensors can be tightly adhered on human skin, such as the armpits and nose (Figure 8c). To show the mechanical robustness of the stretchable sensors, the device was stretched from 0~90% and the typical transfer curves were overlapped (Figure 8d). The extracted on-current and off-currents, as well as mobility, are unchanged (Figure 8e). As the tensile strain of human skin is less than 30%, the stretchable gas sensors are stretched to 30% for 2000 cycles. All the transfer curves show excellent coincidence and stability, indicating the highly mechanical robustness of the stretchable gas sensors under stretching (Figure 8f). Typically, polymer semiconductors have large intermolecular spacings, for example, 16.91 Å for P3HT [68], 25 Å for PCDTPT [69], and 26 Å for PIDTBT [70]. Such intermolecular spacings are much larger than the size of gas molecules (e.g., 5 Å for TMA), enabling sufficient gas sensing even in unstretched polymer semiconductor films. The lubrication effect of side chains, the flexibility of long main chains, and the inhomogeneous hardness distribution caused by the incomplete crystallization of polymer semiconductors lead to a distinctive strain dissipation mechanism when the device is stretched. As a result, the PIDTBT OFET-based sensors exhibit strain-insensitive gas sensing properties. In addition, the different degree of crystallinity stretchable semiconductors, including indacenodithiophene-benzothiadiazole (PIDTBT), diketo-pyrrolo-pyrrole bithiophene thienothiophene (DPPT-TT), and poly[4-(4,4-dihexadecyl-4H-cyclopenta[1,2-b:5,4-b′]dithiophen-2-yl)-alt-[1,2,5]thiad-iazolo[3,4-c]pyridine] (PCDTPT), all show the unchanged gas response signals for the gas of trimethylamine (TMA), NO_2_, and NH_3_ at different strains. Furthermore, the intrinsically stretchable PIDTBT OFET-based sensors exhibit excellent sensitivity to skin-emitted TMA gas, with an ultralow theoretical limit of detection as low as 0.3 ppm, which is sufficient to detect for disease diagnoses, such as trimethylaminuria (TMAU) and bacterial vaginitis (BV) [71], and adjuvant medical therapy.

### 5.2. Stretchable Pressure Sensors Based on OFETs

Pressure is ubiquitous in nature and human activities, arising from forces such as Earth’s gravity and physical contact. Additionally, many physiological processes in the human body generate various pressures, including intraocular pressure and blood pressure. Pressure sensors respond to applied pressure by producing changes in electrical signals, enabling the quantification of mechanical forces through these signals. This remarkable functionality has positioned pressure sensors as essential tools in diverse applications, including human–computer interaction, intelligent prosthetics, and monitoring of human physiological health. Essentially, pressure sensors act as transducers, converting external mechanical stimuli into electrical signals. For the OFET-based intrinsically stretchable pressure sensors, when the sensor is under pressure, the insulating layer becomes thinner, leading to changes in capacitance and modulation of the device’s output current, thereby enabling the highly sensitivity detection of applied pressure.

**Figure 9:**
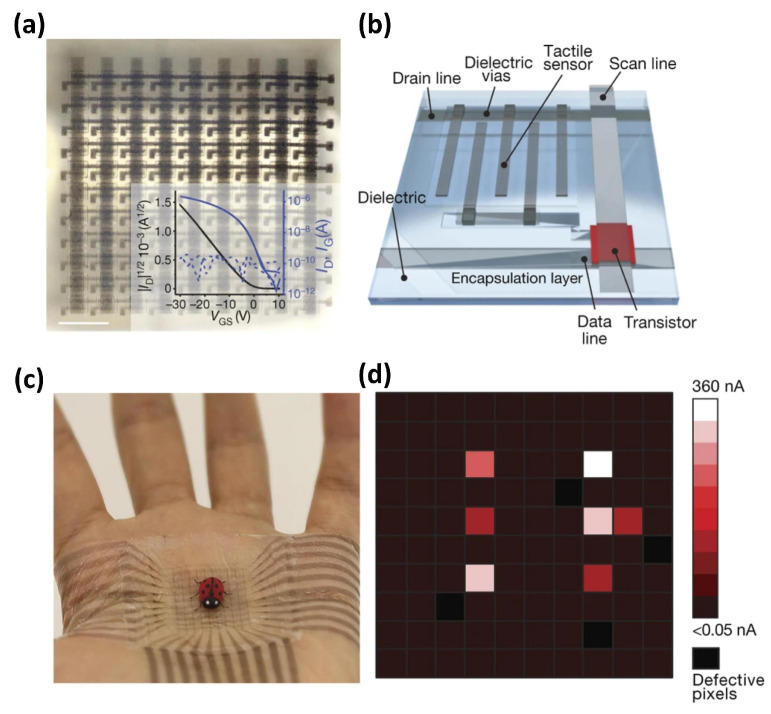
Intrinsically stretchable pressure sensors based on OFETs. (**a**) A 10 × 10 stretchable active matrix transistor array (scale bar: 1 mm). (**b**) A diagram of the tactile sensor array based on the OFET array. (**c**) The array can be adhered to a human palm and accurately detect the position of a synthetic ladybug with six conductive legs. (**d**) Current mapping of the ladybug on the OFET array. Reproduced with permission from [20], copyright 2018, Spring Nature.

Bao et al. [20] reported an intrinsically stretchable 10 × 10 transistor array integrated with stretchable resistive tactile sensors. By incorporating scan and data interconnect lines, they developed an intrinsically stretchable active matrix with a device density of 347 transistors per cm^2^. To demonstrate its application as a multiplexing backplane for skin electronics, they integrated the matrix with a 10 × 10 array of intrinsically stretchable resistive tactile sensors based on interdigitated carbon nanotube electrodes (Figure 9a,b), achieving a resolution of one sensor per 2 mm. The array’s high stretchability enables it to conform seamlessly to the human palm, adapting to its naturally irregular surface and deformation as a secondary skin (Figure 9c). This system accurately detects the position of a small artificial ladybug with six conductive legs via a mapped output of on-current magnitudes from the sensor pixels (Figure 9d). These findings highlight the potential of their intrinsically stretchable active matrix as a backplane for high-resolution touch sensing in highly conformable electronic skins.

**Figure 10:**
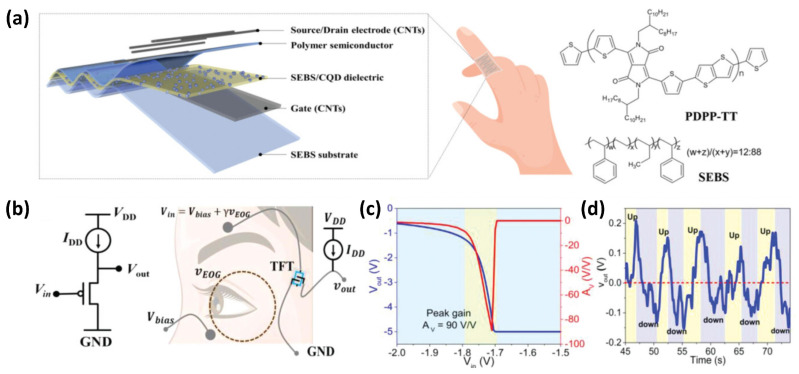
Electro-oculo-gram (EOG) sensor based on stretchable OFETs. (**a**) Diagram of the stretchable OFETs. (**b**) Schematic circuit diagram of the transistor amplifier. (**c**) Output voltage (*V*_out_) and gain (*A*v) curves of the amplifier. (**d**) EOG signals of the amplifier under alternate upward and downward movement of the eyeball. Reproduced with permission from [46], copyright 2018, John Wiley and Sons.

Guo et al. [46] reported a van der Waals-controlling elastomer/carbon quantum high-k hybrid polymer dielectric. The fabricated intrinsically stretchable OFETs (Figure 10a) had a low operating voltage down to −5 V, 100% stretchability, excellent stability, a high on/off current ratio of 10^5^, and steep subthreshold slope of 500 mV dec^−1^. The stretchable low operating voltage and low Young’s modulus enable the device to work safely and adhere to the human body. To detect the weak signals of human physiological signal detection such as electro-oculo-gram (EOG), the OFET is connected with an amplifier (Figure 10b). The two electrodes were positioned, with one on the upper eyebrow and the other on the lower eyelid. The lower electrode was linked to a DC voltage to bias the amplifier, while the upper electrode was connected to the amplifier to output the signal. During eye movement tracking, the amplifier’s input voltage consisted of the DC bias voltage (*V*_bias_, applied beneath the eye) and the AC voltage induced by the eye’s movement (*v*EOG) as *V*_in_ = *V*_bias_ + *γv*_EOG_. Here, *V*_bias_ represents the bias voltage, while *γ* is a coefficient indicating the direction of eyeball movement, with *γ* > 0 and *γ* < 0 corresponding to upward and downward movements, respectively. The ocular electrophysiological signal peaked at approximately 1.5 mV. By analyzing the static transfer characteristics of the OTFT amplifiers, it was determined that a bias current (*I*d) of −70 nA and a supply voltage (*V*_DD_) of −5 V in the saturation region would optimize the amplifier’s gain and cut-off frequency. The amplifier exhibited sharp output voltage (*V*_out_) characteristics, achieving a high gain (A) of up to 90 V/V at an input voltage (*V*_in_) of about −1.7 V (Figure 10c). During both upward and downward eye movements, the optimized OTFT amplifier output alternated significantly from positive to negative potential, with an amplitude exceeding 0.2 V (Figure 10d). This performance outperforms several flexible and rigid OTFT-based amplifiers used for EOG and other electrophysiological monitoring. More importantly, through an analog circuit analysis of the OTFTs, the OTFT-based amplifier demonstrates the first application of stretchable transistors in electrophysiological signal detection, offering a highly sensitive response to subtle eye movements.

### 5.3. Stretchable Strain Sensors Based on OFETs

Strain sensors are devices that convert mechanical stress into electrical signals. They hold significant potential for applications in human skin detection, medical diagnostics, human–computer interaction, wearable devices, and electronic skin. The mechanisms of the reported strain sensors so far are mainly based on resistance, capacitance, and piezoelectricity modes. Compared with these strain sensors, the OFET-based strain sensors integrate signal conversion and amplification into a single device, offering advantages such as fast signal processing, high stability, and low power consumption. Currently, OFET strain sensors primarily use semiconductor layers as the sensitive element. The grain boundaries of semiconductor films change under strain, combined with the application of a gate voltage, allowing the output source–drain current to detect the strain effect.

To date, nearly all strain sensors based on organic semiconductors have utilized polycrystalline thin films as sensing materials and employed three-terminal OFET (organic field-effect transistor) device configurations(Table 2). Strain detection relies on changes in the electrical signals within the thin film. Sekitani et al. first reported the effects of compressive and tensile strain on the electrical properties of pentacene thin-film OFETs [72]. Their experimental results demonstrated that compressive strain increases field-effect mobility, whereas tensile strain decreases it. These changes in electrical properties were attributed to variations in the grain boundary spacing of the thin film under strain [73]. Building on this concept, Seongi’s group developed a thin-film OFET strain sensor and designed a complementary thin-film transistor circuit, achieving an increased sensitivity of 1.51 and successfully enabling the detection of finger movements [74,75].

**Figure 11:**
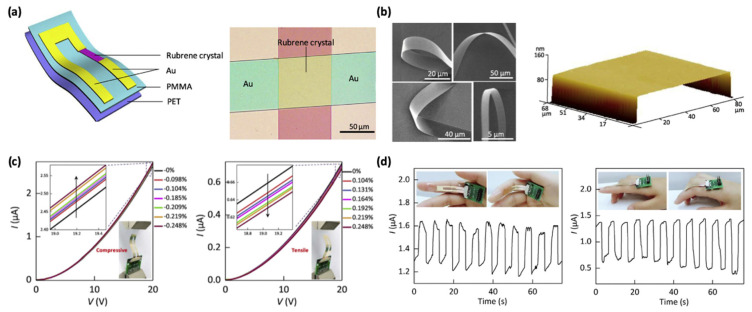
Strain sensors based on OFETs. (**a**) Schematic illustration of rubrene single-crystal OFET strain sensors. (**b**) Optical microscopy image and AFM image of rubrene single crystals. (**c**) *I–V* curves of the rubrene single-crystal device under compressive and tensile strains. (**d**) Real-time current response of the strain sensors during index finger motion under compressive and tensile strains. Reproduced with permission from [76], copyright 2017, IEEE.

Unlike thin-film OFETs, the changes in the electrical properties of single-crystal OFETs under strain are primarily due to variations in molecular spacing. Current research on strain effects in single-crystal OFETs mainly focuses on how strain influences basic field-effect performance. Figure 11a,b presents the transfer characteristic curves of rubrene micro- and nanoscale single-crystal OFETs under compressive and tensile strains. The source–drain current (*I*_SD_) exhibits opposite responses to compressive and tensile strains, where *I*_SD_ increases under compressive strain and decreases under tensile strain [76]. Notably, all devices show a consistent trend in current changes under these strain conditions.

Figure 11c illustrates the relative changes in source–drain current and the mobility of rubrene single crystals under compressive and tensile strains. As depicted, rubrene single crystals demonstrate an approximately linear relationship between strain and changes in both source–drain current and mobility, indicating that variations in *I*_SD_ are driven by mobility changes under strain. The effect of bending on the mobility of organic single-crystal transistors arises from shifts in molecular spacing. Rubrene molecules are connected by relatively weak π bonds, which confer distinct mechanical properties. Compared to inorganic semiconductors bound by covalent or ionic bonds, rubrene single crystals possess a low Young’s modulus and excellent mechanical flexibility. Mechanical compression reduces the molecular spacing in rubrene single crystals, thereby enhancing device mobility, whereas tensile strain increases molecular spacing, resulting in reduced mobility.

To confirm the potential application of rubrene single-crystal field-effect strain sensors in wearable electronics, Figure 11d examines the device’s ability to sense finger-bending motions. The device was attached to a human index finger and placed on both the inner and outer sides of the finger, with *V*_S__D_ and *V*_G_ fixed at −20 V. Under the compressive and tensile strains induced by finger bending, the source–drain current *I*_SD_ increased and decreased rapidly. Furthermore, *I*_SD_ consistently returned to its initial value after each bending and releasing motion cycle, demonstrating the excellent stability of the single-crystal device. These results highlight the potential of organic single-crystal strain sensors for applications in human motion detection and personal health monitoring.

**Table 2:** Comparison of reported strain sensors based on OFETs.

	Sensitive Layer	Strain	Ref.
	Pentacene	1.2%	[77]
	Pentacene	1.5%	[72]
	Pentacene	1%	[78]
	Pentacene	1%	[79]
	Pentacene	>2 mm	[80]
	Pentacene	1 mm	[81]
	Rubrene	0.4%	[76]
Single crystal	C10-DNTT	2.9%	[82]
	Cupc	1.5%	[83]

### 5.4. Stretchable Proximity Sensors Based on OFETs

Recent advancements in robotic systems, prosthetics, and wearable medical devices have driven significant efforts toward the development of highly sensitive skin-mountable sensors. Among the various sensing functionalities, proximity sensing is particularly critical due to its role in enhancing safety measures and enabling non-invasive medical diagnostics and therapeutic applications [84,85]. Proximity sensors, as non-contact sensing devices, can detect the presence and movement of objects without physical contact. Currently, proximity sensors are primarily classified based on their detection principles, including triboelectric [86], magnetic induction [87], capacitive [88], and OFETs [89]. Among them, OFET-based proximity sensors have garnered increasing attention from researchers due to their low cost, ease of fabrication, and potential for widespread application in various fields. OFET-based proximity sensors exhibit higher sensitivity and greater resolution, which are crucial for practical applications in areas such as bionic robot behavior control and security defense. The operating principle of OFET-based proximity sensors is based on the current enhancement effect induced by the “gate” voltage. When an insulating object with a trace charge approaches, the electric field generated by the charge acts as a “gate” voltage, effectively modulating the carrier concentration in the organic semiconductor. This leads to a change in the sensor’s current, enabling the detection of the insulating object. Stretchable proximity sensors based on OFETs exhibit low Young’s modules and high conformability to human skin. However, stretchable proximity sensors based on OFETs have rarely been reported.

**Figure 12:**
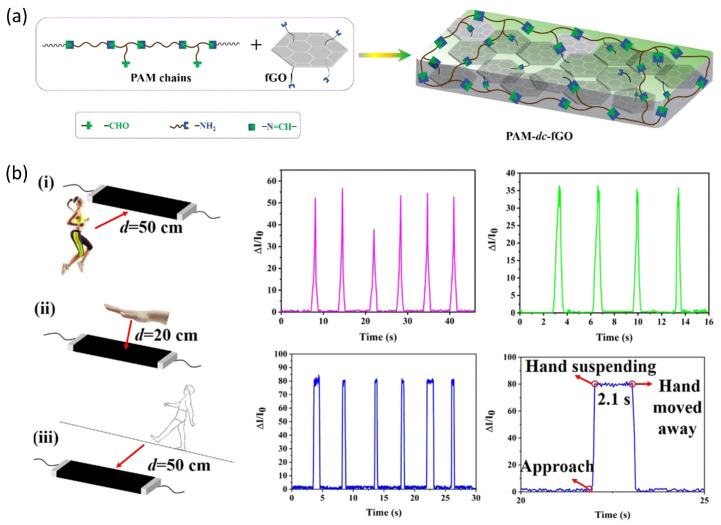
Stretchable proximity sensors based on OFETs. (**a**) Schematic fabrication procedures of PAM-dc-fGO conductive films. (**b**) Schematic illustration of the test methods, including (i) stomping or jumping, (ii) hand movements, and (iii) walking back and forth on a straight line. Reproduced with permission from [90], copyright 2018, ACS publishing.

Kong reported [90] reported a conductive film (PAM-dc-fGO) by combination of polyazomethane (PAM) with functionalized graphene oxide (fGO) (Figure 12a). The obtained conductive films exhibit high mechanical robustness at a stretchability of up to 275% and low Young’s modules of 0.71~4.23 MPa. The fabricated proximity sensors show an ultrahigh gauge factor (GF) of 641 and can precisely detect weak signals from the human body. This enables the advancement of applications in remote monitoring. In such systems, the human body, air layer, and conductive film together form a classical organic field-effect transistor (OFET). When the human body performs activities such as stomping, jumping, or walking, the trace charge induced by the body generates an electric field, which acts as a “gate” voltage. This effectively modulates carrier transport in the conductive film channels, resulting in significant changes in the device current and enabling the remote monitoring of human motion. In addition, the sensors exhibit a high detection distance of about 100 cm (Figure 12b).

### 5.5. Stretchable Temperature Sensors Based on OFETs

Stretchable temperature sensors based on OFETs are emerging as a significant innovation in skin-like electronics. These sensors operate by detecting changes in charge carrier mobility within the organic semiconductor layer, which vary with temperature, allowing for precise temperature sensing. Their advantages include exceptional flexibility and stretchability, enabling integration into wearable devices and curved surfaces. Additionally, they are lightweight, cost-effective, and can be fabricated using scalable techniques like printing. Their high sensitivity and fast response times make them suitable for a wide range of applications, from health monitoring to smart textiles, positioning them as a promising technology for the future of skin-like electronics. The recent progress in intrinsically stretchable organic semiconductor materials, particularly organic polymers, has further enhanced the potential of OFETs for applications in skin-like sensing [91,92].

**Figure 13:**
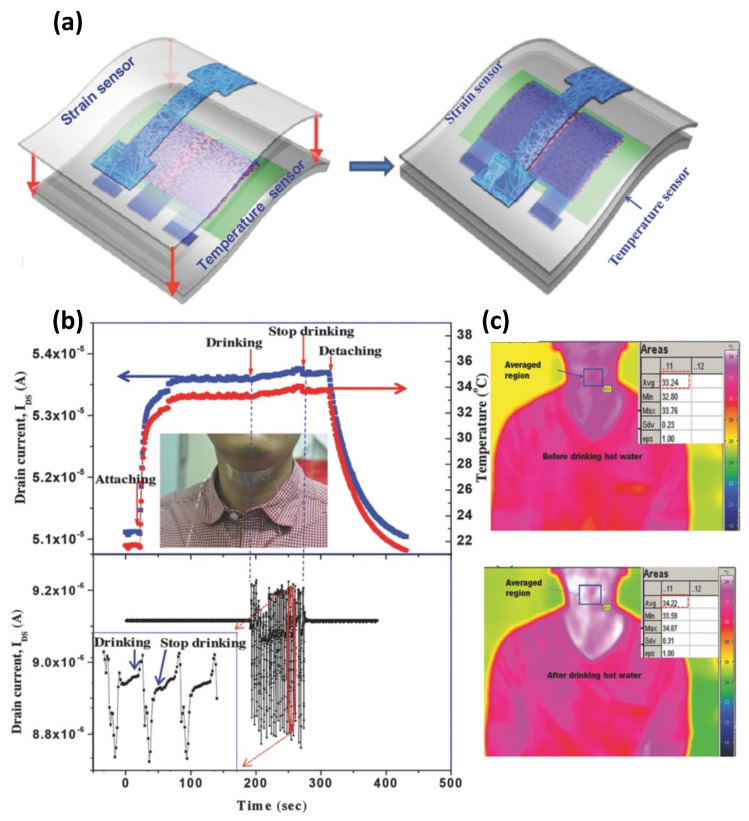
(**a**) Schematic structure of the stretchable temperature sensor. (**b**) Monitoring neck skin temperature and muscle movement. (**c**) IR thermograms of the neck before and after drinking hot water. Reproduced with permission from [93], copyright 2018, John Wiley and Sons.

Table 3 lists the reported stretchable temperature sensors based on OFETs. Lee et al. [93] reported the transparent and stretchable temperature sensor based on OFETs. Inserting conductive and temperature-responsive reduced graphene oxide (RGO) nanosheets into a polyurethane (PU) matrix as the temperature-sensing layer improves temperature sensitivity (Figure 13a). The stretchable temperature sensors exhibit 70% stretchability and a high temperature sensitivity of 1.34% resistance change per °C. Even when the device was stretched to 30% for 10,000 cycles, the responsivity remained nearly unchanged. In addition, the stretchable temperature sensor can successfully measure the human skin temperature. The normal skin temperature of the subject‘s neck is around 33.2 °C (Figure 13b). After drinking hot water, the skin temperature increased to 34.1 °C, which coincides with the calibrated IR camera.

Bao et al. [95] utilized CNTs as the electrodes, SEBS as the substrate and dielectric, and poly(diketopyrrolopyrrole-[3,2-b]thieno[2′,3′:4,5]thieno[2,3-d]thiophene]) (PDPPFT4) and poly(isoindigo-bithiophene) (PII2T) with 70% weight of SEBS as the semiconductor layers. Due to the charge transport mechanism in organic semiconductors and variations in their activation energy, the intrinsically stretchable temperature sensor element achieves an accuracy within 1 °C with uniaxial strains from 0 to 30 %.

Yu et al. [96] reported stretchable temperature sensors based on poly(3-hexylthiophene)/polystyrene-block-poly(ethylene-ran-butylene)-block-polystyrene (P3HT/SEBS) semiconductor composite film OFETs. The device can be stretched to 50% and adhered to robotic fingers to detect the temperature of the human body from 24 °C to 45 °C.

**Figure 14:**
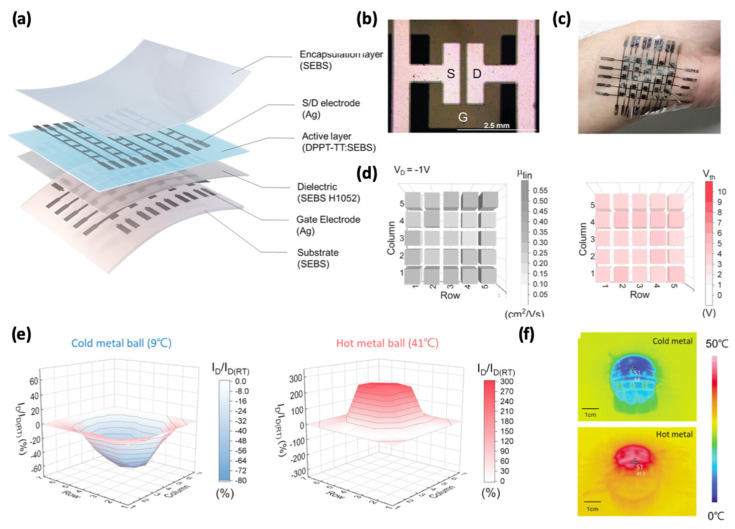
(**a**) Schematic structure of a stretchable temperature sensor based on an OFET array, with SEBS serving as the encapsulation layer, dielectric layer, and substrate; Ag serving as the source, drain, and gate electrodes; and DPPT-TT/SEBS as the active layer. (**b**,**c**) Optical and digital images of the stretchable temperature OFET array conforming to human skin. (**d**) Mobilities and threshold voltages of the OFET array. (**e**,**f**) Three-dimensional NS-current mapping and thermographic images of the OFET array temperature sensor on cold and hot metal balls. Reproduced with permission from [98], copyright 2024, John Wiley and Sons.

Recently, Oh et al. [98] reported a low-power-consumption and highly temperature-sensitive stretchable 5 × 5 OFET array as a skin-like temperature sensor (Figure 14a,b). The device demonstrates ultralow power consumption (<1 nW), high stretchability (100%), high temperature sensitivity (9.4% °C^−1^), and strong adhesion to the human wrist (Figure 14c). The transistor array operated successfully, with minimal variations in mobility and threshold voltage (Figure 14d). To illustrate the sensor’s high temperature sensitivity, a cold (9 °C) and a hot (41 °C) ball were placed on the transistor array. Figure 14e shows the 3D NS-current mapping, which corresponds closely to results from a commercial infrared (IR) imaging camera (Figure 14f). The high temperature sensitivity and excellent stretchability enables the device to work for long time in wearable skin-like electronics.

## 6. Conclusions and Future Outlook

In this review, we have introduced the recent progress of intrinsically stretchable OFETs in functional layer materials, sensor mechanisms, and the applications of stretchable sensors based on intrinsically stretchable OFETs. Although great progress has been achieved in stretchable sensors based on intrinsically stretchable OFETs, many problems still remain to be resolved for the further development of stretchable sensors.

(1) **Designing intrinsically stretchable polymer semiconductors**. As mentioned above, the lack of intrinsically stretchable semiconductors has seriously hindered the development of intrinsically stretchable OFETs and OFET-based sensors. In addition, the poor electrical properties under large tensile strains present another significant challenge for intrinsically stretchable OFETs.
(2) **Improving stability**. There are significant challenges related to the stability of stretchable sensors based on OFETs at long times and strain cycles. As usage time extends, the material may undergo structural changes or degradation, leading to a decline in device performance. Specifically, repeated stretching and compression can impair the device’s electrical properties, manifested as reduced mobility and changes in threshold voltage. Additionally, environmental factors such as temperature and humidity significantly impact device performance. High temperatures may accelerate the decomposition or phase transition of organic materials, while excessive humidity can alter the conductivity of the charge transfer channel and even cause device failure. The combined effects of these factors may limit the long-term stability and reliability of stretchable OFET sensors in practical applications.
(3) **Decreasing the operational voltage**. The currently reported intrinsically stretchable OFETs mainly utilize low-dielectric-constant elastomers, such as poly(dimethylsiloxane) (PDMS), polystyrene-block-poly(ethylene-ran-butylene)-block-polystyrene (SEBS), poly(methyl methacrylate)-poly(*n*-butyl acrylate)-poly(methyl methacrylate) (PMMA-PnBA-PMMA), and perfluoropolyether diols methacrylate (PFPE-DMA). These materials lead to high operational voltages (42~100 V) to overcome the high contact resistance and parasitic capacitance. Reducing the operational voltage to an absolutely safe level for humans (<12 V) is critical for improving energy efficiency, enhancing convenience, and ensuring wearable safety.
(4) **Multifunctional and multimodal sensors**: The next-generation stretchable sensors require multiple sensing capabilities, such as gas sensors, pressure sensors, strain sensors, proximity sensors, and temperature sensors, integrated into a single device. These integrated sensors not only reduce the number of separate sensors and supporting devices in a system, simplifying the electronic circuits and obtaining multiple signals simultaneously, but they also significantly improve sensor performance and application value by combining multiple functions and signal processing capabilities. In the fields of wearable devices, medical health, intelligent robotics, and environmental monitoring, their advantages provide strong support for the realization of more efficient and intelligent detection solutions, representing one of the key directions for the future development of sensing technology.

## Figures and Tables

**Table 1:** Comparison of reported intrinsically stretchable OFETs.

Dielectric	Dielectric Constant	*V*_GS_ (V)	Semiconductor	Electrode	*Μ* (cm^2^ V^−1^ s^−1^)	Stain (%)	Ref.
PDMS	2.7	−60	DPP-polymer	PEDOT/CNT	0.6	100	[18]
PDMS	2.7	−80	P3HT/PDMS	PEDPT:PSS/LiTFSI	0.17	100	[47]
PDMS	2.7	−60	CP/DPP-TT	CNT	0.85	100	[48]
PDMS	2.7	−60	PIDTBT	CNT	1.8	100	[49]
PDMS	2.7	−60	PIDTBT	PEDOT:PSS/CNT	1.84	100	[50]
PDMS	2.7	−60	F4-TCNQ/DPPT-TT	CNT	1.03	100	[51]
PDMS	2.7	−60	PIDTBT	PEDOT:PSS/CNT	1.81	100	[52]
PDMS	2.7	−60	DPP-8TVT	CNT	0.35	100	[53]
PDMS	2.7	−60	PDPP-C4PH	CNT	0.5	50	[54]
PDMS	2.7	−50	PIDTBT	PEDOT:PSS/CNT	2.98	100	[55]
SEBS	2.1	−80	DPPT-TT/SEBS	CNT	0.59	100	[19]
SEBS	2.1	−60	FT4-DPP/PEO	CNT/EGain	0.78	100	[56]
SEBS	2.1	−60	29-DPP-SVS/SEBS	CNT	1.11	100	[20]
SEBS	2.1	−100	C12-DPP	CNT	0.463	100	[57]
SEBS	2.1	−80	DPPDTSE/ SEBS	CNT	1.5	100	[22]
SEBS	2.1	−80	BA-DPPT-TT	CNT	0.5	100	[58]
SEBS	2.1	−60	DPPT-TT/SEBS	Ag	0.28	100	[59]
SBES	2.1	−40	DPPT-TT/SEBS	CNT	0.7	100	[60]
SEBS	2.1	−50	PCDTBT/SEBS	PEDOT:PSS/CNT	0.021	100	[61]
SEBS	2.1	−50	PDVT-10/SEBS	CNT	2.74	150	[62]
SEBS/ CQD	4.1	−5	PDPP-TT/SEBS	CNT	0.6	100	[46]
PVDF-HFP/PVP	9.7	−3	Pse-DPP	AgNW	0.1	100	[31]
NBR/ SEBS/ OTS	28	−3	P-29-DPPDTSE/ SEBS	CNT	2.0	100	[63]
NBR/ SEBS	28	−5	CNT	CNT	20	100	[64]
PUU	12.3	−8	CNT	CNT	10	50	[65]
PUU	53.31	−10	CNT	CNT	76.8	50	[66]
PUU	13.5	−10	PIDTBT	PEDOT:PSS /CNT	1.39	100	[15]

**Table 3:** Comparison of reported stretchable temperature sensors based on OFETs. “/” represents this paper not mentioned this parameter.

Dielectric	Semiconductor	Electrode	Stretchability	Temperature (°C)	Temperature Sensitivity (% °C^−1^)	Ref.
PDMS	R-GO/PU	PEDOT:PSS/PUD	50%	30~80	1.34	[93]
SEBS	SWCNT	SWCNT	60%	15~55	/	[94]
SEBS	PDPPFT4/SEBS PII2T/SEBS	CNTs	30%	25~55	−2.89 −4.23	[95]
PVDF-HFP/[EMIM]^+^[Otf]^−^	P3HT/SEBS	AuNPs-AgNWs/ PDMS	50%	24~45	−7.73~−6.75	[96]
APTES-PDMS	P3HT-NFs/PDMS	PEDOT:PSS	30%	27~45	−0.647	[97]
SEBS	DPPT-TT	Ag	100%	0~50	9.4	[98]

## Data Availability

Data are contained within the article.
